# Commentary: The clinical activity of nurses in general practice

**DOI:** 10.1177/17449871261452914

**Published:** 2026-06-29

**Authors:** Elizabeth Halcomb

**Affiliations:** Professor of Primary Health Care Nursing, University of Wollongong, Wollongong, Australia

**Commentary to:** Clinical scope and workforce contribution of advanced nurse practitioners in general practice: a cross-sectional study (Keye, 2026).

This cross-sectional descriptive study by Keye (2026) addresses a key gap in the international literature around the role of nurse practitioners, and indeed nurses, in general practice. Although the employment of general practice nurses internationally has grown exponentially in recent decades, this has not been accompanied by robust workforce research. In particular, there is very limited data about the clinical activity of general practice nurses or nurse practitioners, the types of presentations they see, the care that they provide or the volume of services delivered. In one if the first international attempts to measure clinical activity, Keye (2026) sought to capture the clinical activity of a group of Irish General Practice Advanced Nurse Practitioners (GP ANP) across a single week. Data sought to explore consultation types, care complexity and clinical outcomes. Although prior research has highlighted the need to better understand nurses’ contributions to general practice care, few international studies have captured this kind of data about nurse activity in general practice (Beattie et al., 2026).

Keye (2026) report using a structured electronic form to collect data. However, there is limited detail provided around the design process used or the final tool composition. Using technology to facilitate in practice data collection is an important strategy to minimise participant burden. In Australia, the Occasions of Care Explained and Analysed (OCEAN) study has recently been launched to examine the workforce and clinical activity of several professions working in general practice, including nurses and nurse practitioners (https://ocean.sydney.edu.au/). Although based on the highly successful general practitioner paper-based study, Bettering the Evaluation and Care of Health (BEACH), OCEAN uses a sophisticated online data collection platform, rigorously co-designed with academics, clinicians and professional stakeholder groups. In clinical research, it is essential to minimise participant burden to maximise engagement from busy clinicians. Including clinicians in co-designing data collection tools and undertaking testing during development, ensures that the final tools can better balance the data that needs to be collected to satisfy the research aims with the burden placed on participants.

In this study, Keye (2026) captured data on 1659 consultations undertaken nationally by 20 GP ANP/Nurse Practitioner Candidates. This represents a substantial body of previously hidden data about the contribution of nurse practitioners to general practice care. The estimation that participants represented approximately 40% of the Irish GP ANP workforce demonstrates a high level of engagement and willingness on the part of nurses to contribute to gathering evidence about their practice. The volume of data implies that the methods of data collection were suitable for the clinical context and did not represent an overly burdensome process.

The high level of clinical activity reported in this study demonstrates the substantial contribution of nurse practitioners to Irish general practice and patient care. The 20 participants were reported to have completed 757 episodes of care within a single week. This not only boosts general practice capacity for service delivery but also offers opportunities to meet increasing community demand for care. The growing number of chronic conditions, an ageing population and shortages in the medical workforce are intensifying the need for innovative, team-based approaches to primary care. This study highlights the important role of nurses in such models. Evidence of clinical activity is also a powerful tool to support lobbying to address key barriers to nursing in general practice such as health policy, funding and remuneration (Halcomb and Ashley, 2019).

Although a key message from this study relates to the importance of the GP ANP role in building general practice capacity, it is important that this is understood within the context of general practice multidisciplinary teams. In their 10 building blocks of primary care, Bodenheimer et al. (2014) highlighted that high performing general practices require multidisciplinary teams of clinicians to meet the growing demand for primary care services. Despite the allure and rapid development of team-based general practice services internationally, evidence suggests that collaboration and teamwork do not just happen (McInnes et al., 2017). Therefore, when examining clinical activity, beyond exploring the contribution of individual clinician groups, consideration should be given to patients’ experiences of general practice care from multidisciplinary clinicians. Although understanding the contribution of individual professions is important, so too is understanding the flow of care within the team to optimise patient-experience, resource utilisation and outcomes.

Overall, this study provides valuable clinical activity data about nurse practitioners’ contribution to general practice care. Although these data address a key gap in the literature, further research using robustly designed tools is needed. Additionally, consideration needs to be given to how measures of clinical activity capture the work of various multidisciplinary general practice team members to demonstrate how teamwork optimises care delivery and service access. Within this, Keye (2026) offers meaningful insights and lays a foundation for further research around clinical activity and team-based general practice care.
